# Dr. Bernard M. Byrne

**Published:** 1860-11

**Authors:** 


DR. BERNARD M. BYRNE, Surgeon in the United States Army, died recently at Fort Moultrie, Sullivan's Island, of typhoid fever. By birth, Surgeon Byrne was an Irishman, but he graduated at the University of Maryland. He distinguished himself in his proper capacity, in many of the battles in Mexico, and published a brochure entitled "An Essay to Prove the Contagious Character of Malignant Cholera," which passed through two editions.

